# Nail Gun Injury and Endovascular Repair of Cervical Internal Carotid Artery

**DOI:** 10.7759/cureus.4237

**Published:** 2019-03-12

**Authors:** Grzegorz Brzezicki, Travis E Meyer, Firas Madbak, Jason Widrich, Abbie Jensen

**Affiliations:** 1 Neurosurgery, University of Florida College of Medicine, Jacksonville, USA; 2 Radiology, University of Florida College of Medicine, Jacksonville, USA; 3 Surgery, University of Florida College of Medicine, Jacksonville, USA; 4 Anesthesiology, University of Florida College of Medicine, Jacksonville, USA

**Keywords:** artery, stent, dissection, angioplasty, trauma

## Abstract

A male patient aged 49 years presented to the emergency room after sustaining a nail-gun injury to the left neck (Zone III). Computed tomography (CT) angiogram demonstrated retained nail traversing in close proximity to the left internal carotid artery. Catheter angiogram with three-dimensional (3D) reconstruction revealed partial left internal carotid injury without active extravasation and with preserved flow through the vessel. The nail was removed by gentle traction with the simultaneous deployment of stent-graft across the injured segment. Balloon angioplasty of the stent was performed secondary to endoleak and active extravasation. Complete vessel reconstruction with maintained blood flow was achieved. The patient was extubated the following day and was discharged home on hospital day five without neurological deficits. This case report demonstrates the usefulness of endovascular repair of high cervical arterial injuries with special attention to the unique nature of nail gun injuries.

## Introduction

Penetrating and blunt trauma of the neck has the potential for catastrophic vascular, airway, digestive and nervous system injury. Carotid artery injuries are present in 22% of cervical vascular injuries, of which 20% involve the internal carotid artery and carry a mortality rate up to 31% and up to 23% stroke rate [1]. Injuries in zone one and three are also difficult to manage surgically due to complex anatomy and poor proximal or distal control [2]. These injuries can be managed successfully with stent-grafts with low mortality and morbidity [3]. 

Nail-gun injuries to the head and neck are rare and usually nonfatal [4]. Limited tissue injury should be expected due to the low velocity of the projectile as compared to gunshots or explosive devices [4]. In this case report, we present a unique nail gun injury to the neck zone 3 with an injury to the left internal carotid, which was successfully treated with simultaneous nail removal and endovascular repair with a covered stent. 

## Case presentation

**Figure 1:**
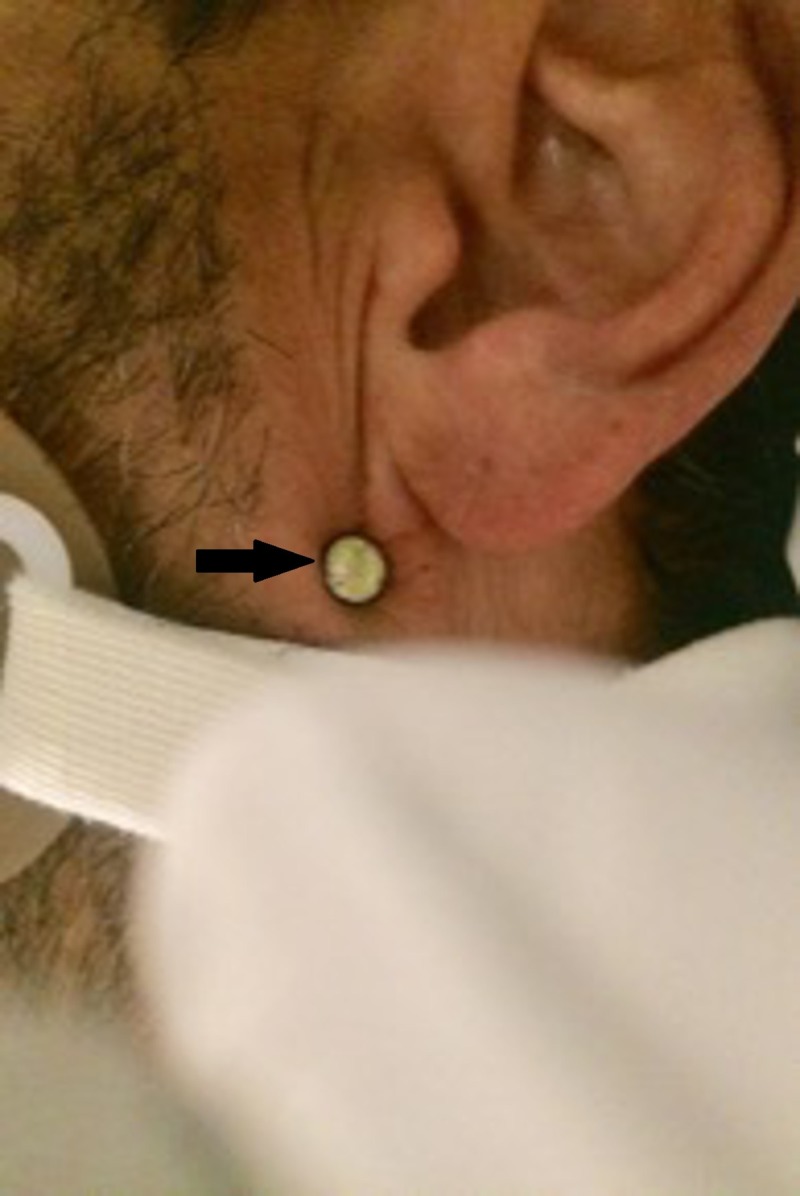
Nail head visible in the left retromandibular region - neck zone 3 injury

A male patient aged 49 years with no previous medical history presented with neck pain from a foreign object. The patient was accidentally struck by a projectile from a nail gun on a construction site. The patient complained of pain along the left side of the neck and inability to swallow. On initial evaluation, patient was neurologically intact with absent Horner's syndrome. There were no signs of active bleeding or hemodynamic instability. The head of the nail was visible behind and above the angle of the mandible at the level of the skin - neck zone 3 (Figure 1, Table 1)

**Table 1:** Anatomic classification of vascular injuries in the neck

Neck vascular injury zones	
Zone 1	From the level of clavicles / sternal notch to the cricoid cartilage
Zone 2	From the cricoid cartilage to the angle of the mandible
Zone 3	From the angle of the mandible to the skull base

**Figure 2:**
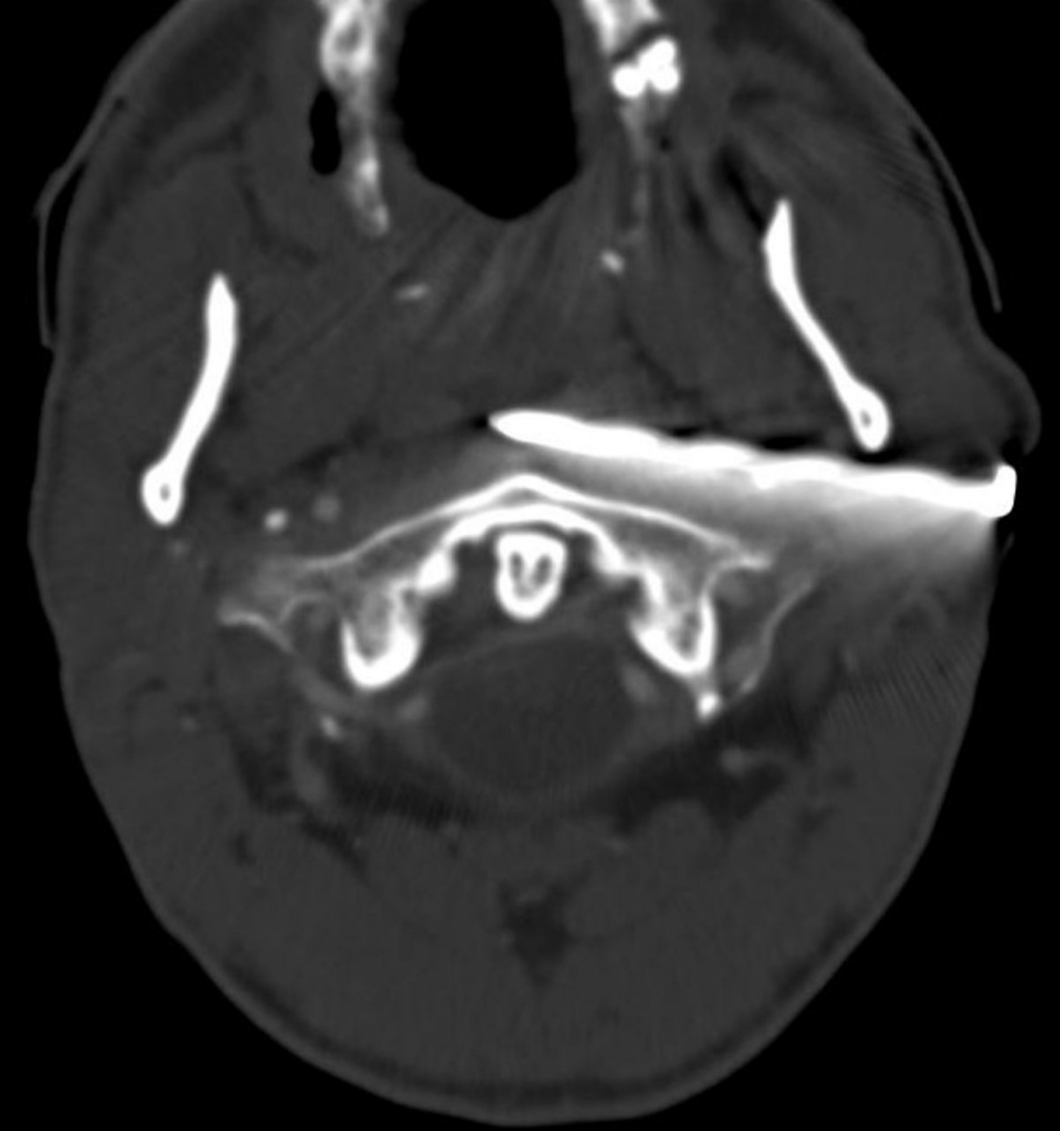
Computed tomography angiography demonstrating nail in the retromandibular region traversing in close proximity to the cervical left internal carotid artery (obscured by metallic artifact from the nail)

The point of the nail was not visible in the oral cavity but was palpable along the oral mucosa along the left side of the oropharynx. The patient was intubated and sedated for airway protection without complications in an outlying emergency room (ER) and triaged to the local Level One trauma center for further management. Upon arrival to our trauma center, a computed tomography (CT) angiography of the neck was obtained which demonstrated the nail traversing in very close proximity to the left internal carotid artery close to the skull base with preserved flow proximal and distal to the nail. There was no active contrast extravasation or definitive presence of a pseudoaneurysm although the evaluation was limited by the beam-hardening artifact from the nail (Figure 2).

**Figure 3:**
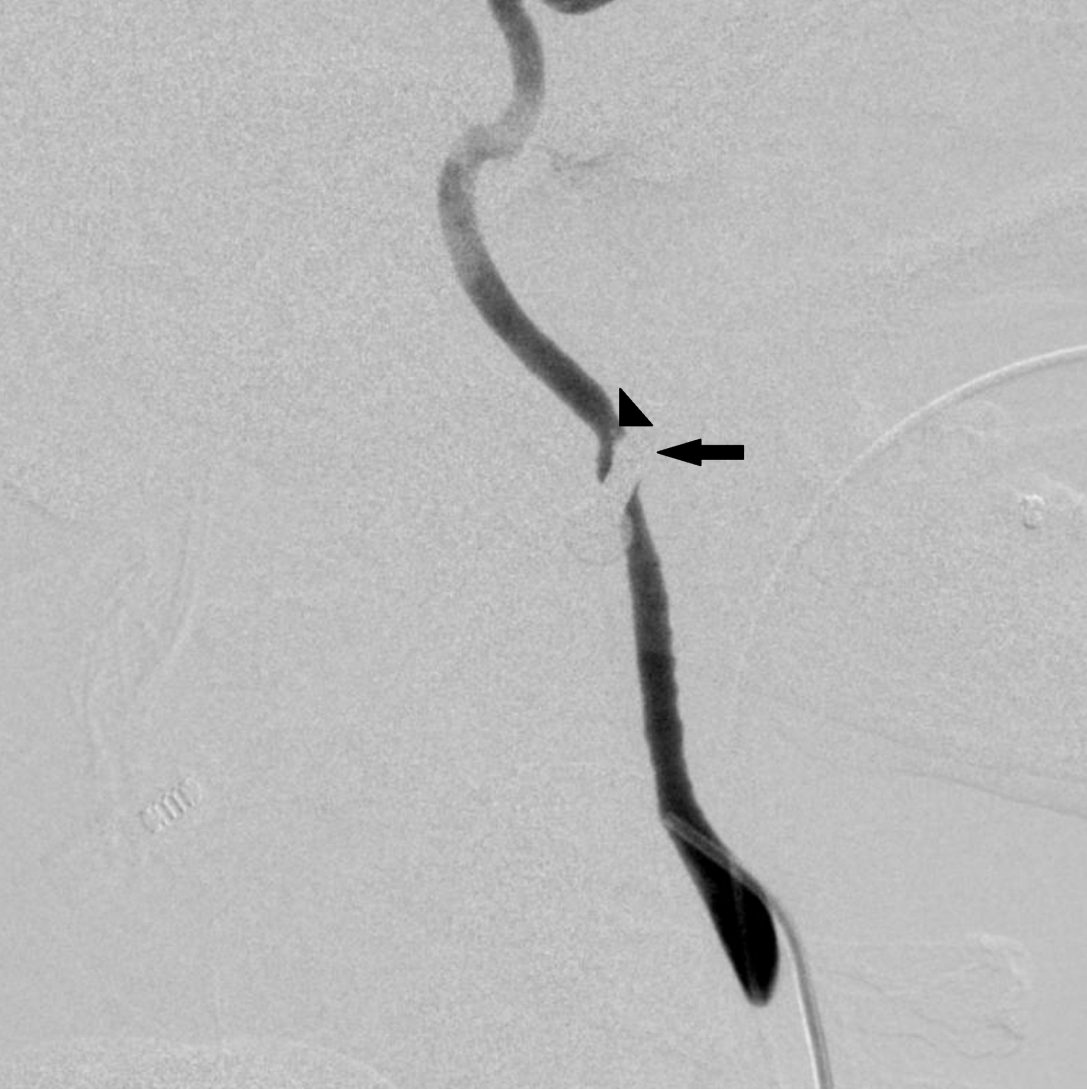
Left internal carotid artery angiogram demonstrating spasm at the level of the nail injury and filling defect consistent with dissection (arrowhead) just distal to the nail (arrow)

 The patient was emergently transported to the interventional radiology suite to determine the status of the left carotid artery and possible endovascular repair. Left common carotid angiography in the neck demonstrated preserved flow through the left internal carotid artery but significant vessel narrowing at the level of the nail (Figure 3).

**Figure 4:**
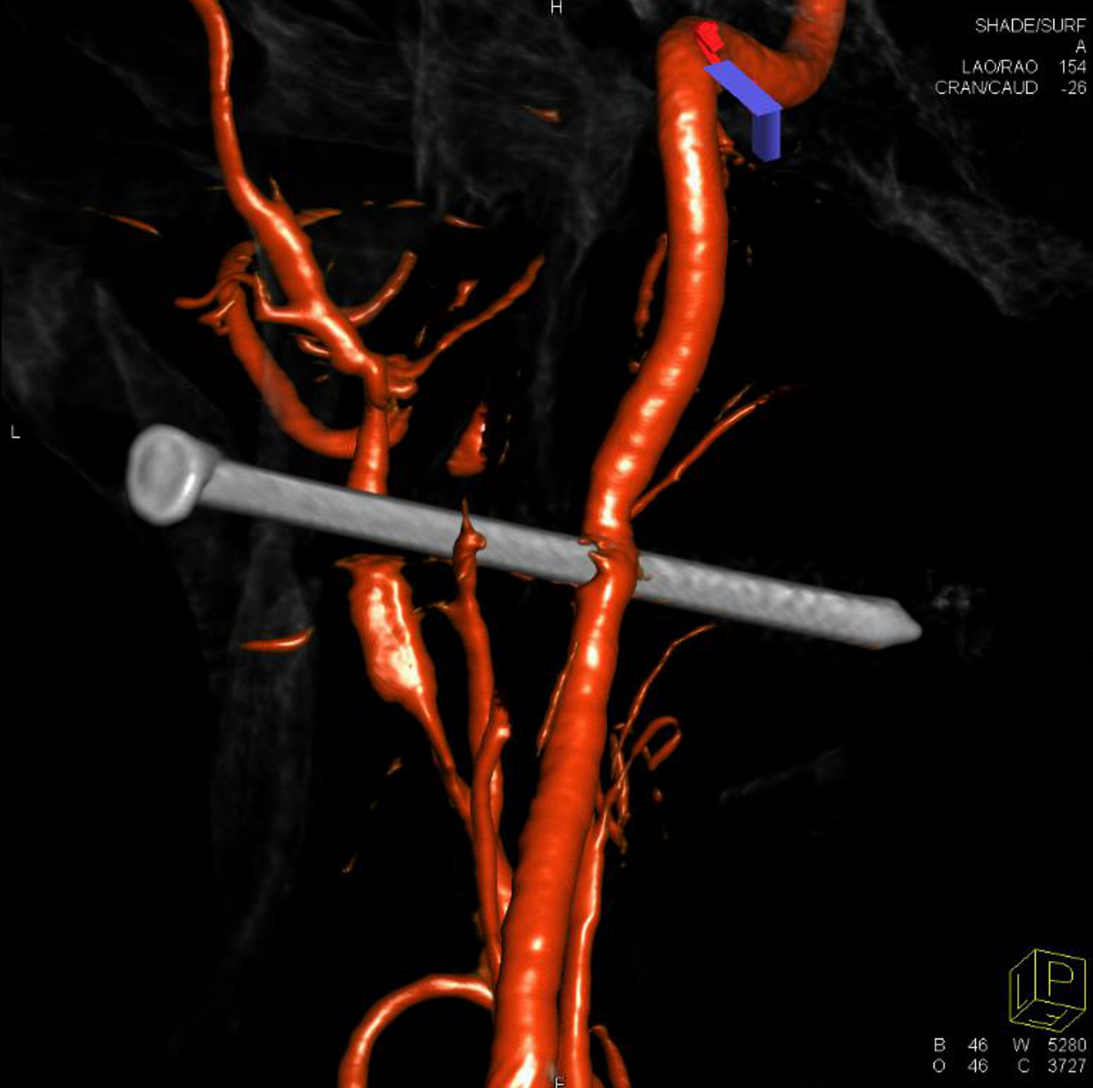
Three-dimensional reconstruction of the left internal carotid artery rotational angiogram demonstrated penetrating nail injury to the carotid artery with preserved flow and vasospasm at the level of injury

Further investigation with three-dimensional (3D) rotational angiography demonstrated small areas of contrast stagnation proximal and distal to the nail with a small dissection flap proximal to the nail, consistent with at least a partial vessel injury (Figure 4).

**Figure 5:**
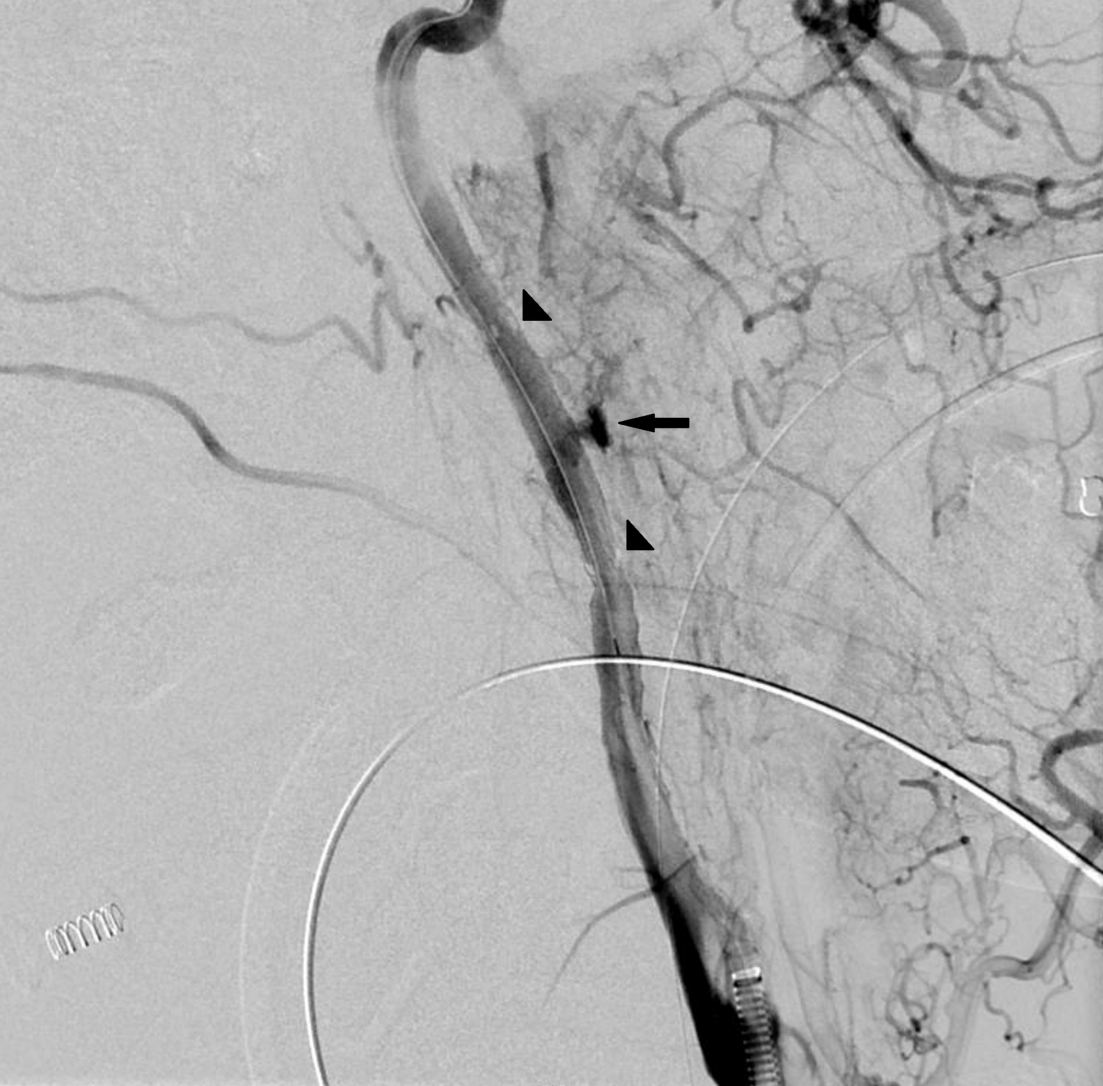
Left internal carotid artery angiogram after deployment of Viabahn stent-graft and removal of the nail demonstrated preservation of flow through the artery and active extravasation from the mid portion of the stent (arrow). Arrowheads indicating proximal and distal stent markers

The intracranial left anterior circulation imaged normally without abnormal vessel dropout. Upon discussion among trauma surgery, oral and maxillofacial surgery and neurointerventional teams, the decision was made to proceed with endovascular repair in conjunction with nail removal. The patient was given 600 mg of clopidogrel and 325 mg of aspirin via orogastric tube and was heparinized to an activated clotting time (ACT) greater than 250. The short 6F sheath was exchanged for 6F Terumo Destination sheath (Terumo Medical, Somerset, New Jersey, USA) and positioned in the distal left common carotid artery. The injury site was crossed with a Synchro-2 0.014 microwire (Stryker, Fremont, California, USA) under the fluoroscopic roadmap. A 5 x 25 mm Gore Viabahn covered stent (W.L. Gore & Associates, Flagstaff, Arizona, USA) was positioned across the level of injury. The nail was removed by gentle traction by trauma surgery with the simultaneous deployment of the Gore Viabahn covered stent into the left internal carotid artery spanning the injured segment. Follow-up angiography demonstrated excellent flow through the stent but with active extravasation secondary to a proximal endoleak related to incomplete apposition of the proximal stent to the vessel wall (Figure 5).

**Figure 6:**
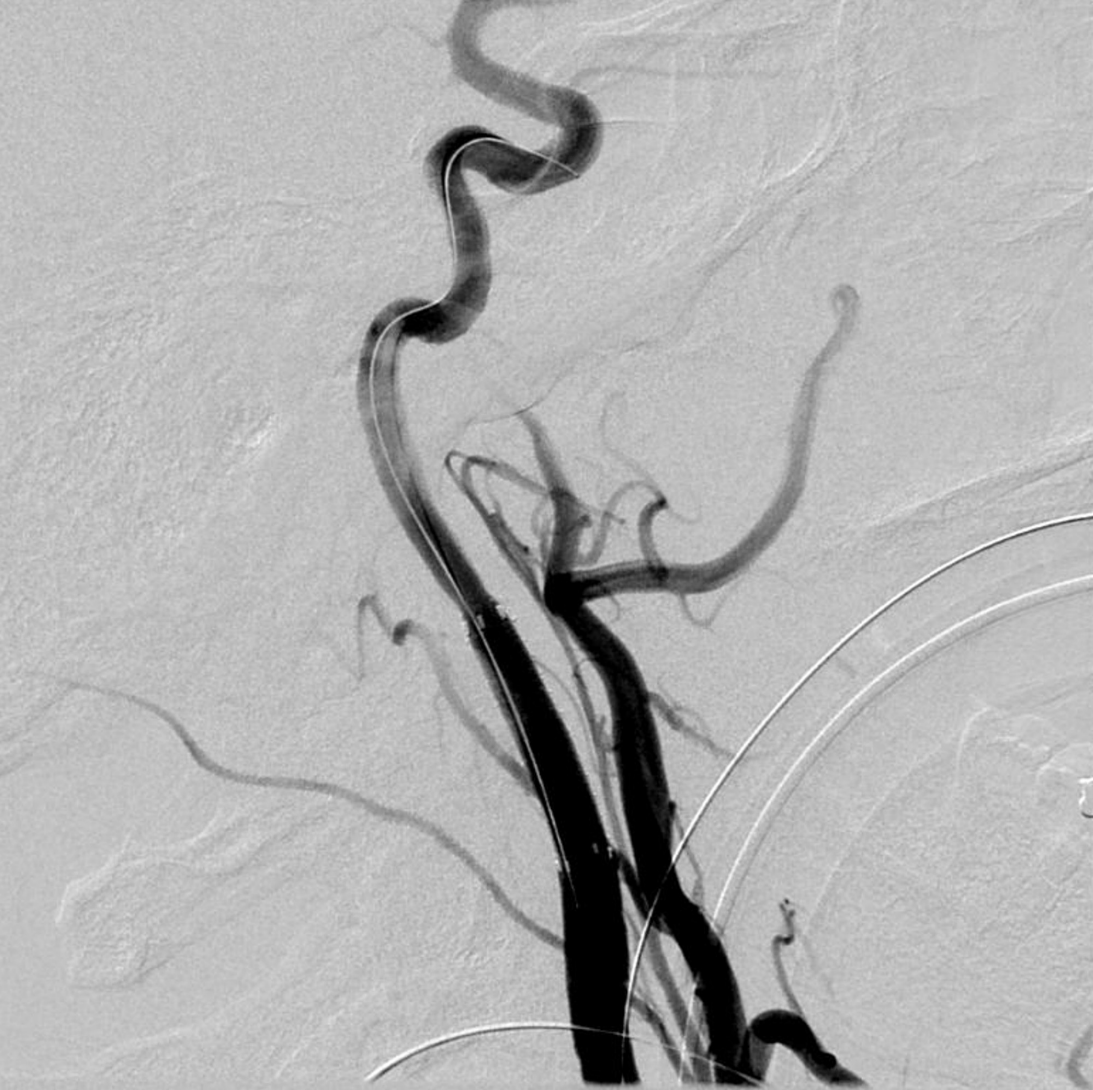
Left internal carotid artery angiogram after balloon angioplasty of the stent demonstrated no further extravasation and better vessel wall apposition

At this time, the anaesthesia team noted blood pooling in the oropharynx. We introduced a 5 x 30 mm Aviator Plus balloon (Cordis, Milpitas, California, USA) and performed angioplasty of the proximal end of the stent. Follow-up angiography demonstrated no active extravasation and excellent flow through the stent (Figure 6).

Cerebral angiography demonstrated no abnormal vessel dropout in the left anterior circulation. There was no further bleeding in the oropharynx or through the tract. The patient was transferred to intensive care unit (ICU) for further care. 

**Figure 7:**
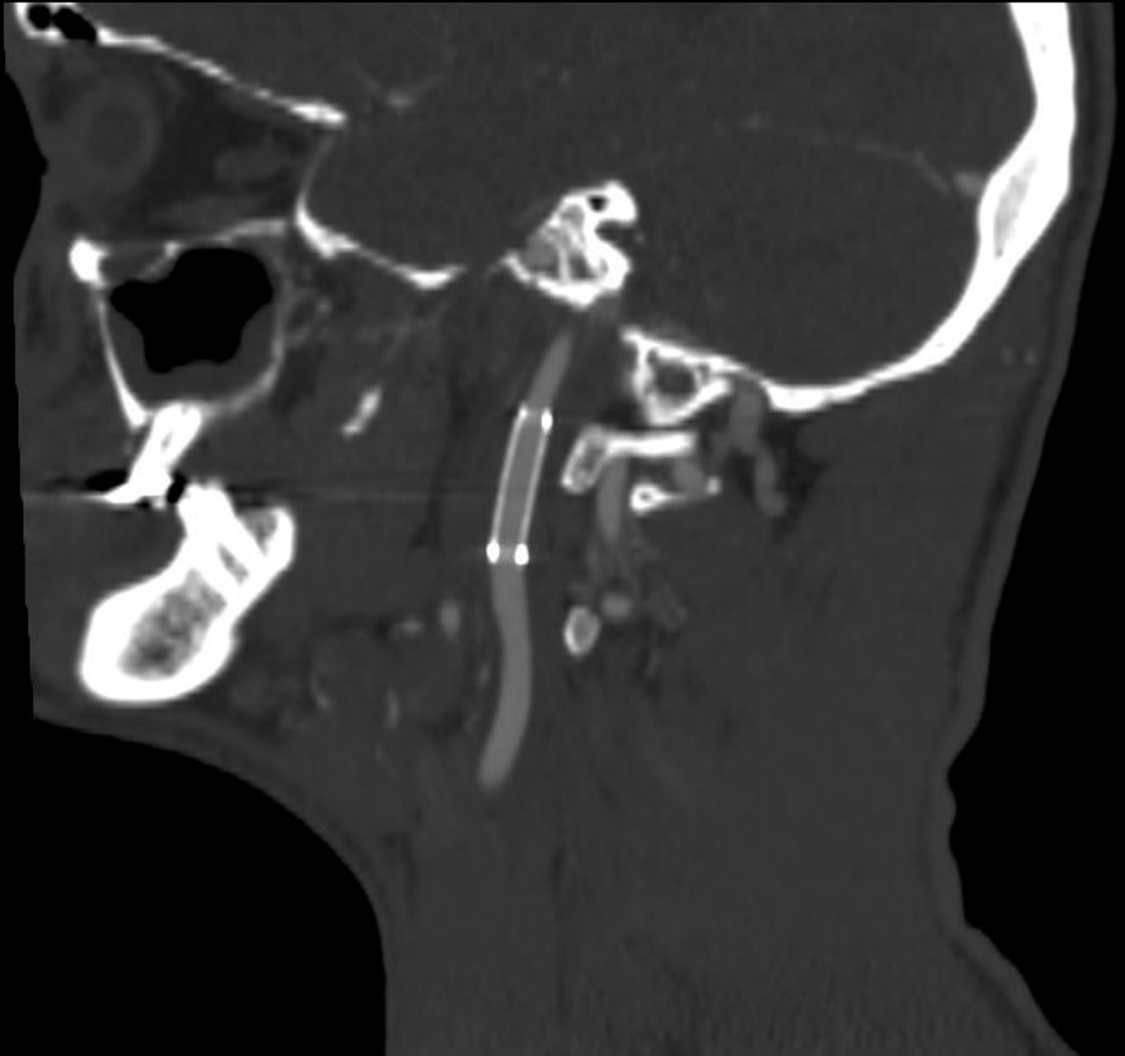
Computed tomography angiography neck 24 hours after endovascular repair showed patent stent and no evidence of pseudoaneurysm formation

On a postoperative day one, the patient was extubated without incident. No neurological deficits were noted. The patient was continued on 325 mg of Aspirin and 75 mg of clopidogrel daily. The patient was cleared for an oral diet. Follow-up CT angiogram demonstrated patency of the left internal carotid stent and no pseudoaneurysm formation (Figure 7). The patient did receive tetanus boosters and antibiotics as part of the initial management to prevent secondary infection. The patient was discharged to home on hospital day five. We planned to follow up the patient with CT angiogram at six weeks after the procedure. Unfortunately, we were not able to obtain the imaging as the patient was out of state and was uninsured. Per phone conversation the patient was doing very well and did not report any symptoms consistent with stroke or transient ischemic attack (TIA) at two months after the procedure. The patient stopped his clopidogrel one week after the procedure due to financial reasons but continues on the aspirin regimen.

## Discussion

Penetrating injuries to the neck involving the carotid artery have been described both in civilian and military settings [1-3]. Management of these injuries has varied from emergent open surgery, endovascular intervention or observation with serial imaging. When compared to gunshot or blast injuries, nail-gun injuries are unique as they are low velocity, small calibre, penetrating injuries, which cause very limited tissue damage. As such, they share some similarities in the extent of tissue injury and possible neurological complications with the inadvertent arterial placement of central venous catheters, which can be successfully treated with covered stents [4].

There are only a few reports describing cases of nail gun injury to the head and neck [4-6]. Buchalter et al. described three cases of nail injuries to the head and skull base. All of the patients underwent a diagnostic cerebral angiogram to determine possible vascular involvement. One patient suffered an injury to the internal maxillary artery at the pterygomaxillary space and was treated with embolization. This enabled for simple nail removal rather than extensive craniofacial exploration with anticipated significant blood loss [4]. In our case we were able to perform a successful endovascular reconstruction, allowing for safe nail removal without zone 3 neck exploration. Endovascular carotid sacrifice was contemplated as a bailout option if open or endovascular reconstruction was not possible. Adequate balloon test occlusion would have been difficult given emergent situation and intubated patient. Selvanathan et al. described a case of self-inflicted nail-gun injury to the skull base with penetration into the carotid canal [7]. Initial angiography did not clearly indicate injury to the carotid artery and the injury was confined to the carotid canal therefore, the nail was removed by gentle traction. Postoperative angiography demonstrated a three-millimeter internal carotid artery pseudoaneurysm which was treated conservatively with aspirin. The pseudoaneurysm was present on three-month follow up magnetic resonance angiography (MRA) and the patient was scheduled for a repeat angiogram. It is worth mentioning that our angiographic findings were very similar to this case and only the 3D rotational angiogram demonstrated very subtle signs indicating vessel injury.

The use of covered stents in extracranial carotid artery injuries was described before, both in adult and pediatric populations [3, 8]. A case series by du Toit et al. includes 19 patients with stab injuries to the carotid artery in zone one and three including five cases of stab injuries to the internal carotid artery, which were treated with covered stent placement and mean follow-up of 44 months. There was one death related to a pre-existing large infarction with associated brain edema and two stent-graft thrombosis including one symptomatic case. Of note, there was poor compliance with antiplatelet medications as well as mostly aspirin monotherapy in this cohort. The authors recommended stent-grafting as a primary method of treatment for zone 1 and 3 carotid injuries and gave serious consideration for use in all zones [3]. Herrera et al. report a case series of 36 patients with carotid injuries treated with different endovascular techniques (stents, covered stents, coils) including seven patients with stab wounds. The internal carotid artery was involved in 39% of these injuries and one patient presented with active bleeding. Covered stent deployment resulted in immediate angiographic occlusion and no immediate complications. The use of noncovered stents resulted in pseudoaneurysm occlusion in 77.8% at six months. Out of 14 patients treated with stents who had follow-up imaging, there was one asymptomatic stent occlusion (patient decided to stop antiplatelet medications) and one mild intimal hyperplasia. In conclusion, the authors recommend using noncovered stents due to the lower risk of restenosis and similar efficacy in lower grade injuries and covered stents or vessel sacrifice for more extensive injuries [8]. In our case, the use of a noncovered stent would likely result in ongoing active extravasation necessitating rescue maneuvers and possible vessel sacrifice. In a review paper by Alaraj et al., which included 150 patients implanted with 164 covered stents for carotid and vertebral artery pathologies including carotid blowout syndrome, arteriovenous fistulas and pseudoaneurysms, the technical success rate was 98.2%. Immediate complications occurred in 9.1% of procedures including two strokes and six transient ischemic attacks. 8.3% of patients developed asymptomatic occlusion of the stented vessel. In-stent stenosis was observed in another four cases [9]. End-stent stenosis is more common for covered stents compared to conventional stents and can be more pronounced at a bend or kink [10]. The duration of dual antiplatelet is highly debatable, although six months’ duration is recommended by some authors given the large area of synthetic material exposed to circulation [9]. In our case the covered stent was deployed in a straight segment of the vessel and we planned for six months of dual antiplatelet therapy. Unfortunately patient discontinued clopidogrel one week after the procedure due to financial reasons but did not report symptoms of thromboembolic complications up to two months after the procedure.

## Conclusions

The exact degree of vascular injury inflicted by nail gun might be difficult to grade prior to removal of the nail due to limited tissue disruption caused by this type of projectile. A 3D rotational angiogram might provide additional clues indicating higher grade injury necessitating vessel repair. Endovascular repair with stent-grafts appears to be the optimal method of managing arterial nail-gun injuries specifically in difficult to access zones one and three providing immediate vessel reconstruction without morbid surgical intervention.

## References

[1] du Toit DF, van Schalkwyk GD, Wadee SA, Warren BL (2003). Neurologic outcome after penetrating extracranial arterial trauma. J Vasc Surg.

[2] Reva VA, Pronchenko AA, Samokhvalov IM (2011). Operative management of penetrating carotid artery injuries. Eur J Vasc Endovasc Surg.

[3] du Toit DF, Coolen D, Lambrechts A, de V Odendaal J, Warren BL (2009). The endovascular management of penetrating carotid artery injuries: long-term follow-up. Eur J Vasc Endovasc Surg.

[4] Buchalter GM, Johnson LP, Reichman MV, Jacobs J (2002). Penetrating trauma to the head and neck from a nail gun: a unique mechanism of injury. Ear Nose Throat.

[5] Alberico G, Bucci I, Ciarelli F, De Giorgio G, D'Artista D, Ciccarelli O (1997). An unusual case of nail gun injury: penetrating neck wound with nail retention in the right pleural cavity. J Trauma.

[6] Lee BL, Sternberg P (1996). Ocular nail gun injuries. Ophthalmology.

[7] Selvanathan S, Goldschlager T, McMillen J, Campbell S (2007). Penetrating craniocerebral injuries from nail-gun use. J Clin Neurosci.

[8] Herrera DA, Vargas SA, Dublin AB (2011). Endovascular treatment of penetrating traumatic injuries of the extracranial carotid artery. J Vasc Interv Radiol.

[9] Alaraj A, Wallace A, Amin-Hanjani S, Charbel FT, Aletich V (2011). Endovascular implantation of covered stents in the extracranial carotid and vertebral arteries: case series and review of the literature. Surg Neurol Int.

[10] Willfort-Ehringer A, Ahmadi R, Gschwandtner ME, Haumer M, Lang W, Minar E (2002). Single-center experience with carotid stent restenosis. J Endovasc Ther.

